# Online Pharmacy Adoption and Consumer Perception: A Systematic Review of Existing Literature

**DOI:** 10.7759/cureus.97319

**Published:** 2025-11-20

**Authors:** Shamsuzzaman Ansari, Sudhinder S Chowhan, Sarthak Sengupta

**Affiliations:** 1 School of Pharmaceutical Management, Indian Institute of Health Management Research (IIHMR) University, Jaipur, IND; 2 Department of Pharmaceutical Management, Institute of Health Management Research, Bangalore, IND; 3 Department of Health and IT, Institute of Health Management Research, Bangalore, IND

**Keywords:** consumers’ characteristics, consumers’ perception, customers’ perception, customers’ satisfaction, e-pharmacy, internet pharmacy, mail order pharmacy, online pharmacy, online pharmacy business model, web-based pharmacy

## Abstract

Online pharmacy, also known as e-pharmacy, is an online platform that enables customers to buy medicinal products and services, ensuring convenient delivery to their homes in a short timeframe. The adoption of online pharmacies has increased significantly in recent years, primarily driven by lower prices offered by online pharmacies, the absence of specific medications in conventional markets, and the convenience aspects, such as the ease of access and decreased reliance on physical pharmacy visits. This review paper aimed to thoroughly explore the existing literature regarding online pharmacies, with particular attention to three key areas delineated in the current literature. These areas encompass the following: types of online pharmacy business models; consumer characteristics; and consumers’ attitudes and perceptions toward online pharmacies. The research methodology included a comprehensive literature search on online pharmacies from January 2020 to mid-January 2024 using SCOPUS, PubMed, and Web of Science. Studies published in English within the defined timeframe were considered for inclusion, whereas those focusing on other than consumers’ perceptions and attitudes or not presenting full-length content were excluded. The preliminary search identified more than 1,700 articles, which were then systematically filtered down to 24 articles selected for final review. This review analysis suggests that women are frequent online pharmacy customers, with awareness levels varying across genders and demographics. Middle-aged individuals, particularly between 35 and 74, show a strong inclination toward online medication purchases, driven by urbanization and internet access. Education and income also influence consumer behavior, albeit to varying degrees across regions and products. Convenience, discounts, and product availability emerge as primary drivers for online pharmacy adoption, with factors like home delivery and 24/7 accessibility enhancing convenience. Trust remains pivotal, with offline pharmacies favored for reliability due to perceived risks. Recent studies spotlight consumer priorities such as logistics and pricing, highlighting areas for online pharmacy service enhancement. In conclusion, this systematic exploration of consumers' attitudes and perceptions toward online pharmacies reveals a dynamic landscape influenced by technological advancements and changing consumer behaviors.

## Introduction and background

The rapid advancement of technology, particularly the internet, has profoundly transformed human behavior and communication with our surroundings, continuing to expand swiftly to even the most remote parts of the world [1,2]. The continual transformation of the digital landscape and technological advancements have brought about substantial modifications in consumption patterns, business strategies, and promotional methods [3]. In the United States, research indicates that 56% to 79% of internet users look for health information online [4]. India has the second-largest internet-using population in the world, second only to China [5]. A significant portion of the Indian population, constituting 52% or 759 million individuals, is now a regular internet user as of 2022, as per the research released by the Internet and Mobile Association of India (IAMAI) and Kantar in May 2023. The projections suggest that by 2025, this number is anticipated to reach 900 million. Among the overall count, there are 399 million people residing in rural India, whereas 360 million people live in urban India. Rural areas in India will account for 56% of new internet users by 2025. Urban India witnessed a moderate 6% rise in 2022, with approximately 71% internet penetration. The majority of the overall gain in numbers can be credited to rural India, which enjoyed a growth rate of 14% over the preceding year[6].

In the realm of online transactions, encompassing e-commerce activities, India has surpassed the United States. Almost 346 million Indians engage in online transactions, which encompass activities such as e-commerce and digital payments. The number mentioned surpasses the estimated total inhabitants of the United States, which is currently 331 million [7]. The utilization of e-commerce in the healthcare sector marked the inception of online pharmacies, which commenced in the United States in 1999. This encompassed the online sale of both over-the-counter (OTC) and prescription medications [8]. E-pharmacy, also known as online pharmacy, is an online platform that enables customers to buy medicinal products and services, ensuring convenient delivery to their homes in a short timeframe [9,10]. E-pharmacy business segment gained prominence through entrepreneurs striving to provide affordable and quality healthcare to people. The preference for e-pharmacy surged from 23% in 2013 to approximately 59% in 2018 [11]. E-pharmacy’s utility peaked during the lockdowns induced by the COVID-19 pandemic, particularly when there was a surge in the demand for medicines. The government even classified its operation as an "essential service." Numerous research papers published previously have underscored significant factors influencing the inclination of individuals to embrace online pharmacies. These factors encompass price discrepancies, the absence of specific medications in conventional markets, and the convenience aspects, such as the ease of access and decreased reliance on physical pharmacy visits [8,12]. According to Statista, the global E-pharmacy market is expected to reach a staggering amount of USD 52.51billion by 2024. Additionally, analysts foresee a compound annual growth rate (CAGR 2024-2028) of 11.57%, leading to an estimated market size of USD 81.37 billion by 2028. A few pharmaceutical companies are also collaborating with online pharmacy platforms to facilitate the direct distribution of their products to consumers. For instance, in March 2024, Eli Lilly forged a partnership with Amazon's pharmacy division to enable home delivery of medications for conditions such as diabetes, migraines, and obesity, including the GLP-1 weight loss medication Zepbound. In January 2024, Eli Lilly launched LillyDirect, a direct-to-consumer service offering telehealth consultations, pharmacy services, and the option to obtain certain medications directly from the company through an online channel [13]. As per Sastasundar Ventures, the Indian online pharmacy market amounted to US$345 million in 2021 and is projected to expand at a compound annual growth rate of 22%. The dominant players in the Indian E-pharmacy sector include TATA 1 mg, PharmEasy, Flipkart Health Plus, Apollo 247, and Netmeds, among various others [14].

There are no specific, dedicated regulations for online pharmacies in India. In India, pharmacies adhere to the Drug and Cosmetics Act of 1940, the Drugs and Cosmetics Rules of 1945, the Pharmacy Act of 1948, and the Indian Medical Act of 1956. The regulations pertaining to e-commerce are outlined in the Information Technology Act of 2000. In August 2018, the Union Health Ministry introduced draft regulations with the objective of overseeing the online distribution of medicines, aiming to guarantee the accessibility of genuine drugs via legitimate online channels. Nevertheless, the South Chemists and Distributors Association opposed this draft regulation through a petition. They argued that the proposed rules, which neglected the health risks associated with unregulated online medicine sales, were being advanced in clear violation of the law. Fast forward to March 2023, the ministry informed the Delhi High Court that they were currently contemplating a proposal to establish regulations for e-pharmacies, acknowledging the need for additional time in the process [15].

Recent systematic review studies, summarized in Table 1, have explored various aspects of online pharmacies, including their types and characteristics, the quality of pharmaceutical drugs purchased online, consumer profiles, motivations for buying prescription medicines over the Internet, and factors influencing consumers’ intentions and behaviors toward online medicine purchases.

**Table 1:** Recently published systematic review articles

Review Article	Publication Year	Scope of the Article
What influences consumers' online medication purchase intentions and behavior? A scoping review [16]	2024	Examines all qualitative and quantitative studies of consumers’ online medication purchase intentions and behavior.
Reasons that lead people to buy prescription medicines on the internet: a systematic review [17]	2023	Explores evidence on the reasons that drive people to purchase prescription medicines via the Internet.
Illicit online pharmacies: a scoping review [18]	2023	Identifies key themes arising in the IOP literature, main gaps where additional research is necessary, and directions for future research.
Online pharmacies selling prescription drugs: systematic review [19]	2022	Focuses on type and characteristic of online pharmacies; quality of pharmaceutical drugs purchased online; and characteristics of consumers purchasing pharmaceutical drugs from online pharmacies.

As the research study of online pharmacies has experienced significant expansion in recent years, it is imperative to present the most up-to-date findings concerning these platforms, concentrating primarily on recent publications. This review paper aimed to thoroughly explore the existing evidence regarding online pharmacies, with particular attention to three key areas delineated in the current literature. These areas encompass the following: types of online pharmacy business models; consumer characteristics; and consumers’ attitudes and perceptions toward online pharmacies.

## Review

Methodology

Search Strategy

The literature search covered all articles published related to online pharmacies from January 2020 until mid-January 2024. The search was performed on three electronic databases, namely, SCOPUS, PubMed, and Web of Science. The search terms listed in Table 2 were performed on three electronic databases, namely, SCOPUS, PubMed, and Web of Science. These terms were either used alone or in combination with Boolean operators.

**Table 2:** Keywords

Search Terms
Online pharmacy
E-pharmacy
Internet pharmacy
Digital pharmacy
Tele pharmacy
Web-based pharmacy
Virtual pharmacy
Mail order pharmacy
Cyber pharmacy

Inclusion and Exclusion Criteria

The inclusion and exclusion criteria for studies in this review have been listed in Table 3.

**Table 3:** Inclusion and exclusion criteria

Inclusion Criteria	Exclusion Criteria
The review considered papers published between January 1, 2020, and January 15, 2024.	Reviews, research notes, letters to the editor, and case reports were excluded as they did not meet the inclusion criteria for this comprehensive review.
In the final analysis, only those articles were included that discussed online pharmacy business models, consumer characteristics, and consumers’ attitudes and perceptions toward online pharmacies.	
Full-length articles written exclusively in the English language were considered for the review. This criterion was applied to ensure a comprehensive and standardized examination of the relevant scientific literature.	

Article Filtering Mechanism

**Figure 1:**
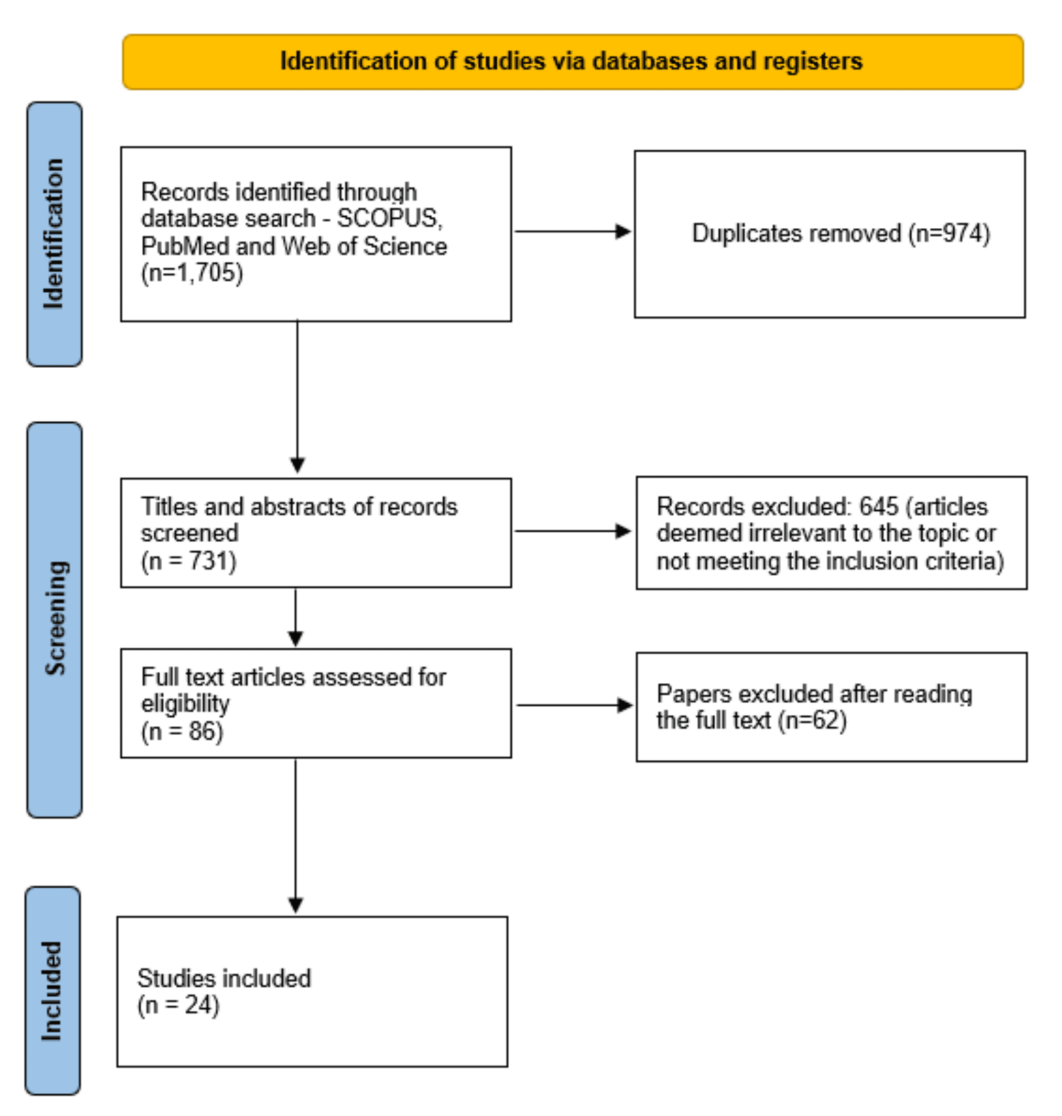
Preferred Reporting Items for Systematic Reviews and Meta-Analyses (PRISMA) flow diagram

The initial database search across all three databases yielded a total of 1,705 articles. After removing 974 duplicate records, 731 unique articles remained for screening. Titles and abstracts of these 731 articles were reviewed, leading to the exclusion of 645 articles that were either irrelevant or did not meet the inclusion criteria. The remaining 86 articles were assessed in full text, following which 24 articles were selected for the final analysis. An overview of the selection process is presented in Figure 1, following the Preferred Reporting Items for Systematic Reviews and Meta-Analyses (PRISMA) guidelines.

Results

Online Pharmacy Business Models

Of the 24 selected articles, four described different business models of online pharmacies. Details of these studies are presented in Table 4. Online pharmacy companies have adopted different models, such as the stock-based online pharmacy model, the marketplace-driven online pharmacy model, and the generic e-commerce marketplace model. In the stock-based model, the e-commerce organization oversees both the products and the operations, directly delivering items to customers. A comprehensive inventory of medications distributed across different regions/individuals is maintained. In a marketplace-driven approach, online pharmacies serve as intermediaries connecting consumers with retailers. Operating as a consolidator, the online pharmacy establishes an electronic link that connects clients with drug suppliers. This type of online pharmacy platform encompasses licensed pharmacies, cataloguing their products. Generic e-commerce marketplaces, commonly referred to as generic digital platforms, provide a diverse array of products such as hardware, design items, furniture, home goods, and beauty products. It's important to note that advertising services or promoting prescribed medications is strictly prohibited on these platforms [11,20,21]. Another study distinguishes between provider-facing and consumer-facing online pharmacies, predominantly focusing on the latter. Consumer-facing models predominantly fall into two categories: inventory-based establishments fulfilling e-orders and marketplace models facilitating connections between consumers and traditional pharmacies via mobile applications. The inventory-based approach includes purely online operations as well as hybrid models integrating both online and brick-and-mortar setups. Notably, the COVID-19 pandemic has prompted brick-and-mortar pharmacy chains to establish online platforms, while general online retailers are expanding their inventory to include medicines, and new market entrants are emerging [22].

**Table 4:** Review of literature on online pharmacy business models

Study Title	Author(s)	Year	Country	Study Design	Journal
The rise of E-pharmacy in India: benefits, challenges, and the road ahead[11]	Dcruz et al.	2022	India	Qualitative	Indian Journal of Pharmacology
Has the outbreak of the Coronavirus pandemic impacted the online pharmacy in serving the nation or capitalization of business opportunities in India? [20]	Sanawane and Mahajan	2020	India	Qualitative	Journal of Management Research
Online pharmacy: is risk or benefit to patient? [21]	Dasgupta et al.	2023	India	Qualitative	World Journal of Pharmaceutical Research
When technology precedes regulation: the challenges and opportunities of e-pharmacy in low-income and middle-income countries [22]	Miller et al.	2021	Kenya, India, Nigeria	Interview	BMJ Global Health

Consumers’ Characteristics

Seven articles out of 24, listed in Table 5, concentrated on the characteristics of consumers purchasing drugs from online pharmacies. A 2022 survey in Russia indicated that women and older individuals tend to buy drugs more frequently. Older respondents commonly purchase drugs, likely due to the prevalence of chronic illnesses. Younger respondents show higher demand for dietary supplements, cosmetics, and hygiene products, possibly reflecting an active lifestyle and responsibility for caring for elderly relatives or loved ones [23]. In a cross-sectional survey-based study conducted in Saudi Arabia in 2023, it was found that individuals under the age of 40 were more inclined to be familiar with online pharmacies and to make purchases from them compared to older participants. Additionally, men exhibited lower awareness of online pharmacies and, if they were aware, were less prone to purchasing from them compared to women. Those residing with children were more likely to be familiar with online pharmacies than those without.

**Table 5:** Review of literature on online pharmacy consumers’ characteristics

Study title	Author(s)	Year	Country	Study design	Journal
Problems of purchasing pharmacy products through online orders [23]	Soboleva et al.	2022	Russia	Survey	Journal of Advanced Pharmaceutical Technology and Research
Public awareness of online pharmacies, consumers’ motivating factors, experience and satisfaction with online pharmacy services, and current barriers and motivators for non-consumers: the case of Saudi Arabia [25]	Almohammed et al.	2023	Saudi Arabia	Survey	Saudi Pharmaceutical Journal
A preliminary study to evaluate the behavior of Indian population toward E-pharmacy [8]	Bansal et al.	2022	India	Survey	Indian Journal of Pharmacology
Trends and characteristics of the US adult population's behavioral patterns in web-based prescription filling: national survey study [26]	Yang et al.	2021	US	Survey	Journal of Medical Internet Research
Online pharmacy: customer profiling [27]	Cherecheș et al.	2021	Romania	Interview	Acta Medica Marisiensis
Consumers’ usage and adoption of e-pharmacy in India [28]	Srivastava et al.	2021	India	Survey	International Journal of Pharmaceutical and Healthcare Marketing
Covid 19 pandemic impacts on online pharmacy and offline pharmacy with reference to Mumbai region [29]	Ushir et al.	2022	India	Survey	Asian Journal of Pharmaceutical Research and Development

Participants with higher levels of education were more inclined to make purchases from online pharmacies [24,25]. A research study conducted on the Indian populace asserted that awareness of the online availability of medicines is similar among both genders. However, urban dwellers exhibited greater awareness compared to their rural counterparts[26]. Among those who lacked awareness about online pharmacies, the majority were graduates [8]. In a study examining the US adult population’s behavioral patterns in web-based prescription filling, it was found that individuals in the age range of 50 to 64 years, identified as female, of White ethnicity, and possessing higher levels of education, income, and insurance coverage, demonstrated significantly higher likelihoods of utilizing web-based prescription services. Prescription drugs form an essential component of the offerings provided by e-pharmacies.

Furthermore, marital status emerged as a significant determinant, with respondents who were married or cohabiting exhibiting a greater propensity for web-based prescription filling compared to those who were divorced, widowed, single, or never married. Additionally, respondents who had been employed within the past 12 months were more inclined to utilize online prescription services compared to their unemployed counterparts [27]. A Romanian-based study conducted in 2020 reveals that the demographic characteristics of individuals who perceive online alternatives as significant and indispensable align with the hypothesis that early adoption of digital innovations is primarily motivated by education and higher income levels. Specifically, individuals purchasing medications through online platforms exhibit traits akin to those who responded affirmatively to preceding inquiries: they demonstrate activity, possess elevated educational attainment, and enjoy disposable financial resources [28]. A research study done in Bangalore, India, found that more young people are buying medicines from online pharmacies. Nevertheless, there is no correlation between educational background and gender when it comes to their adoption of e-pharmacies [29].

Consumers’ Attitudes and Perceptions Toward Online Pharmacies

Of the 24 articles, 19 described consumers’ attitudes and perceptions toward online pharmacies, as shown in Table 6. Six of these overlapped, addressing both consumer characteristics and their attitudes and perceptions regarding online pharmacies. The majority of consumers are aware of the existence of online pharmacies for the purchase of medicines[25,30]. However, another study on the Indian population suggests that there is a low level of awareness among people regarding the trustworthy sites for buying medicines [8]. Also, the majority of respondents are not aware of the legal safeguards or rules for purchasing medicines online [30]. Most studies emphasize the primary attributes that draw users to online pharmacies, such as low prices as compared to offline pharmacies, refill reminders, accessibility for persons with disabilities, non-availability of medications in the local market, availability of a large range of products, round-the-clock service, home delivery, and availability of doctors linked with online pharmacies [8,23,25,27-29,31-35].

**Table 6:** Review of literature on consumers’ attitudes and perceptions toward online pharmacies

Study title	Author(s)	Year	Country	Study design	Journal
A preliminary study to evaluate the behavior of Indian population toward E-pharmacy [8]	Bansal et al.	2022	India	Survey	Indian Journal of Pharmacology
Problems of purchasing pharmacy products through online orders[23]	Soboleva et al.	2022	Russia	Survey	Journal of Advanced Pharmaceutical Technology and Research
Public awareness of online pharmacies, consumers’ motivating factors, experience and satisfaction with online pharmacy services, and current barriers and motivators for non-consumers: the case of Saudi Arabia [25]	Almohammed et al.	2023	Saudi Arabia	Survey	Saudi Pharmaceutical Journal
Online pharmacy: customer profiling [27]	Cherecheș et al.	2021	Romania	Interview	Acta Medica Marisiensis
Consumers’ usage and adoption of e-pharmacy in India [28]	Srivastava et al.	2021	India	Quantitative survey-based study	International Journal of Pharmaceutical and Healthcare Marketing
Covid 19 pandemic impacts on online pharmacy and offline pharmacy with reference to Mumbai region [29]	Ushir et al.	2022	India	Survey	Asian Journal of Pharmaceutical Research and Development
A study of consumer perception towards online pharmacy [30]	Inamdar	2021	India	Survey	International Journal of Advance and Innovative Research
Consumer perception towards online pharmacy and offline pharmacy with reference to Mumbai city [31]	Ushir et al.	2022	India	Survey	Asian Journal of Pharmaceutical Research and Development
The effects of online pharmacy on consumer behaviour [32]	Javed and Shaiq	2023	Pakistan	Survey	Propel Journal of Academic Research
Investigating the factors influencing the adoption of online pharmacy in Oman [33]	Sideiri et al.	2021	Oman	Qualitative analysis	Journal of Hunan University Natural Sciences
Patient satisfaction with online pharmacy services [34]	Manoliu-Hamwi et al.	2022	Romania	Survey	E-Health and Bioengineering Conference
Evaluating the frequency, consumers’ motivation and perception of online medicinal, herbal, and health products purchase safety in Saudi Arabia [35]	Alwhaibi et al.		Saudi Arabia	Survey	Saudi Pharmaceutical Journal
Consumer perception towards e-pharmacy in Gujarat [36]	Lakhani and Kumar	2021	India	Survey	PIMT Journal of Research
Understanding the determinants of online pharmacy adoption: a two-staged SEM-neural network analysis approach [37]	Sabbir et al.	2020	Bangladesh	Survey	Journal of Science and Technology Policy Management
Perceived risk and online purchase intention of E-pharmacy: examining the moderating role of online trust in the Indian context[38]	Varghese Assin et al.	2022	India	Survey	Specialusis Ugdymas
Analysis of competitive forces on the performance of indian retail pharmacy: with special reference to online pharmacy as a new entrant [39]	Thakur et al.	2023	India	Survey	Journal of Applied Management-Jidnyasa
Consumers' satisfaction factors mining and sentiment analysis of B2C online pharmacy reviews [40]	Liu et al.	2020	China	Sentiment analysis	BMC Medical Informatics and Decision Making
The voice of drug consumers: online textual review analysis using structural topic model [41]	He et al.	2020	China	Sentiment analysis	International Journal of Environmental Research and Public Health
Customer satisfaction evaluation for drugs: a research based on online reviews and PROMETHEE-Ⅱ method. [42]	Zhao et al.	2023	China	Sentiment analysis	PLoS one

Javed and Shaiq, who conducted a research study in Pakistan, underscored that most individuals recognize the advantages of privacy, contactless transactions, and swift delivery offered by online pharmacies. Consequently, they are drawn toward purchasing medicines online [32]. Consumer purchasing patterns in online pharmacies are also shaped by previous encounters with online pharmacies, familiarity with other e-commerce platforms, and the experiences shared by acquaintances [23,25,29,36,37]. According to a study conducted in Oman, the word-of-mouth (oral suggestion) is a powerful approach to encourage others to utilize the online pharmacy [33]. In a few research studies, consumers have expressed their satisfaction with the flexibility and control they experience when using online pharmacy platforms. They feel empowered by the ability to choose their preferred medication brands and add them to their shopping carts, contrasting with the traditional physical pharmacy setting where the pharmacist typically guides the selection process [23,28].

A research study conducted in India indicates that customers believed that in order to streamline the purchasing experience from online pharmacies, e-vendors should offer guidance throughout the buying process, ensuring it is smooth and hassle-free [28]. The impact of perceived risk on the intention to use online pharmacies among individuals who haven't yet adopted e-pharmacy services in India is noteworthy. The research indicates that various dimensions of risk perception, including financial, physical, and source risks, are significantly inversely related to consumers' inclination to make online purchases from e-pharmacies. A few experts also suggest that the quality of information and user-friendly navigation play key roles in building this trust. Conversely, outdated or inaccurate information could instill doubt and mistrust among customers [28,33,37,38]. According to research carried out, consumers in India tend to trust purchasing medication from physical medical shops over online pharmacies, perceiving them as more reliable compared to internet-based platforms or applications [31]. The primary factors deterring non-consumers from buying from online pharmacies included their preference for visiting community pharmacies and the challenge of discerning between reputable and untrustworthy vendors. A research study conducted by Thakur and Mandhanya concerning the Indian population uncovered that four primary factors-shopping convenience, trustworthiness, the non-prescription drug market, and personalized counselling play a significant role in the proliferation of online pharmacies [39].

In a 2020 publication by Liu et al., a comprehensive examination was conducted to delve into the factors influencing consumer satisfaction within the realm of Online pharmacy reviews for business-to-customer (B2C) transactions. The study sought to analyze the sentiments expressed in these reviews with the ultimate objective of aiding B2C online pharmacy enterprises in pinpointing consumer concerns and facilitating a continuous enhancement of health service levels. The outcomes of this data mining initiative revealed that, in the realm of drug purchases, consumers exhibit the highest level of interest in logistics, closely followed by considerations for drug prices and customer service. Surprisingly, the least attention was devoted to the evaluation of drug effects by consumers. This research sheds light on the nuanced priorities of consumers, offering valuable insights to guide improvements in the services provided by B2C online pharmacies [40]. Another sentiment analysis conducted by He et al. revealed that the expiration date of drugs and after-sales service are the two primary factors contributing to dissatisfaction among consumers of online pharmacies[41]. Additionally, a study by Zhao et al. based on online reviews from Alibaba Health Pharmacy suggests that consumer expectations vary depending on the type of drugs purchased through online pharmacy portals. For instance, when it comes to tonic drugs, customers prioritize "drug cost performance" as the most important factor, while "online customer service" is viewed as a secondary consideration [42].

Discussions

This systematic literature review undertook a comprehensive and up-to-date examination of the scientific literature concerning online pharmacies, employing a focused strategy that encompassed searching three databases, namely, SCOPUS, PubMed, and Web of Science. Our search parameters were confined to articles released between January 2020 and mid-January 2024. During the specified timeframe, twenty-four articles were published that fulfilled the inclusion criteria, with twelve of them being pertinent to India. While there were no restrictions on research methodologies, the articles identified primarily consisted of surveys. The impetus for conducting this new review stemmed from the heightened utilization of online pharmacies in the aftermath of the COVID-19 pandemic. In countries such as India and numerous European nations, the prevalence of online pharmacies was relatively low before the COVID-19 pandemic. Nevertheless, there was a substantial surge in adoption during the pandemic, and this trend has persisted even after COVID-19 [25,27,29,30].

The first objective of this study was to acquire a comprehensive understanding of the existing online pharmacies’ business model. While reviewing published articles, it is understood that there is a lack of a standardized online pharmacy business model, and most companies have adopted models such as the stock-based online pharmacy model, marketplace-driven online pharmacy model, and generic e-commerce marketplace model. The onset of the COVID-19 pandemic spurred traditional brick-and-mortar pharmacy chains to launch online platforms, while online retailers in general are broadening their product offerings to include medications. The second objective of this study was to analyze consumers’ characteristics. Most studies indicate that drugs from online pharmacies are more often purchased by women. A Saudi-based study suggests that men were comparatively less aware of online pharmacies compared to women, while research conducted on the Indian population asserted that awareness of the availability of medicines online is similar across genders. People between the ages of 35 and 74, in contrast to both younger and older demographics, show a greater inclination toward purchasing medications online, largely due to their active lifestyles and the responsibility of caring for elderly relatives and loved ones. Urban populations exhibited higher levels of awareness compared to rural areas, likely attributable to the greater internet penetration in urban settings. As internet usage continues to rise in rural regions, this discrepancy is expected to diminish. As stated in the introduction, rural areas in India are projected to represent 56% of new internet users by 2025, indicating a significant shift toward greater internet access in these regions. The limited awareness of online pharmacies in rural areas might also stem from the educational gap between rural and urban areas, as urban populations typically exhibit higher levels of education compared to rural counterparts [43]. Higher education also correlates with more use of online pharmacies for prescription fulfillment. Consumers taking multiple medications showed a greater inclination toward online pharmacies over offline ones, with higher-income individuals more inclined toward obtaining medications online. However, these trends may not universally apply to all products, medications, or geographic regions. For example, certain research indicates that in India, factors such as age, gender, and educational background show no correlation with the adoption of e-pharmacies for buying medications. This finding is mirrored in a study conducted in 2011 among the Hungarian population as well [44].

The third objective of the study was to analyze consumers’ attitudes and perceptions toward online pharmacies. Most studies highlight convenience, attractive discounts, and the wide availability of pharmaceutical products as primary factors that attract consumers toward online pharmacies. Convenience encompasses factors such as home delivery, expedited delivery, 24/7 availability, user-friendliness, and easy accessibility, among others. Additionally, the option for doctor consultations offered by online pharmacies impacts consumers' intentions and behaviors regarding purchasing medications online. Offline pharmacies can compete with online counterparts by improving consumer convenience offerings. A research study indicates that certain respondents are hesitant to use online pharmacies due to perceived time consumption. To address this concern, e-vendors must ensure swift access to pharmacist, physician, or customer support services during the buying process to prevent potential abandonment. Thus, promoting e-pharmacy as a "time-efficient" platform for purchasing medicines is crucial for e-vendors to encourage positive perceptions [28]. Online pharmacy consumer purchasing behaviors are also influenced by past interactions with online pharmacies, familiarity with e-commerce platforms, and shared experiences from acquaintances. It is crucial to acknowledge the significance of establishing trust in online pharmacies due to the perceived risk associated with online shopping. The findings reveal that various dimensions of risk perception, encompassing financial, physical, and source risks, are notably and inversely related to consumers' willingness to engage in online purchases from e-pharmacies. Many consumers prefer to trust offline pharmacies for medication purchases, viewing them as more reliable compared to internet-based platforms or applications. In the past four years, only three research studies have been published examining consumer sentiments conveyed in online reviews regarding online pharmacies, utilizing mining and sentiment analysis. Findings from one of these studies indicate that consumers prioritize logistics the most when buying drugs, followed by drug prices and customer service, while paying the least attention to drug effects [40]. Consequently, online pharmacies should focus on enhancing their logistics. If they lack a robust internal delivery system, they may need to depend on third-party logistics services. Another sentiment analysis conducted by Zhao et al. based on online reviews from Alibaba Health Pharmacy suggests that consumer expectations vary depending on the type of drugs purchased through online pharmacy portals[42].

Limitations of the Study

This study offers valuable insights into consumers’ characteristics, attitudes, and perceptions toward online pharmacies; however, certain limitations should be acknowledged. First, the timeframe considered was relatively limited, spanning from January 2020 to mid-January 2024, which may have resulted in the omission of more recent or emerging studies in this domain. Second, the search was restricted to only three databases, potentially excluding relevant literature available in other databases such as ScienceDirect or Google Scholar. Lastly, the scope of this review focused primarily on articles addressing online pharmacy business models, consumer characteristics, and consumer attitudes and perceptions. Consequently, studies exploring other relevant aspects, such as regulatory, technological, or ethical perspectives, have not been captured.

Future Research Directions

While numerous research studies have been undertaken recently in India, the majority have focused on tier one cities. However, there is a scarcity of research concerning consumers in tier two and tier three cities in the context of online pharmacy usage. Given the expanding presence of Indian online pharmacies in tier two and tier three cities, it is crucial to comprehend the behavior of consumers from these regions as well. Most of the articles included in the study, which investigated consumer behavior regarding the online purchase of prescription medicines, employed a quantitative methodology, primarily utilizing surveys for data collection. Only three academic papers have delved into examining consumer sentiment perceptions by analyzing online reviews of online pharmacies. It's worth mentioning that these studies have focused solely on Chinese online pharmacy consumers. Notably, there is a lack of research specifically addressing the Indian context or any other countries, highlighting a significant gap in the literature. No research has specifically aimed at conducting a tailored consumer survey solely for college-aged individuals (Gen Z) in India. Additionally, there has not been any investigation into segmenting online medicine purchases based on therapeutic areas.

## Conclusions

In conclusion, our systematic exploration of consumers' attitudes and perceptions toward online pharmacies reveals a dynamic landscape influenced by technological advancements and changing consumer behaviors. The uptake of online pharmacies in India, as well as in other countries, has surged notably during the COVID-19 pandemic, and this trend persists. There is a lack of a standardized online pharmacy business model. This systematic review suggests that women are more frequent purchasers from online pharmacies, with varying awareness levels among genders in different populations. Middle-aged individuals, particularly between 35 and 74, exhibit a higher inclination toward online medication purchases, influenced by urbanization and internet accessibility. However, factors like education level and income also impact consumer behavior, although their significance may vary across regions and products. Consumer perspectives on online pharmacies highlight convenience, discounts, and product availability as key factors of appeal. Factors such as home delivery and 24/7 accessibility contribute to convenience, while options for online consultations influence purchasing decisions. Offline pharmacies can compete by improving convenience offerings. Trust is crucial due to perceived risks, with consumers often preferring offline pharmacies for reliability. Recent studies analyzing online reviews highlight consumer priorities such as logistics and pricing, indicating areas for improvement in online pharmacy services. The study also identified areas lacking in research and delved into prospects for future research endeavors.
